# Impact of a suicide prevention learning module for firearm training courses in Louisiana

**DOI:** 10.1186/s40621-024-00526-0

**Published:** 2024-09-02

**Authors:** Claire Houtsma, Lauren Reyes, Katherine MacWilliams, Gala True

**Affiliations:** 1 Southeast Louisiana Veterans Health Care System, New Orleans, LA USA; 2 South Central Mental Illness Research, Education and Clinical Center, 2400 Canal Street, New Orleans, LA 70119 USA; 3 Louisiana State University Health Sciences Center, New Orleans, LA USA

**Keywords:** Firearm owners, Firearm instructors, Video, Secure storage

## Abstract

**Background:**

Firearm suicide is a leading cause of death in the United States. Suicide prevention experts have advocated for upstream interventions that can be implemented prior to the development of suicidal thoughts, particularly those that focus on lethal means safety (LMS; e.g., increasing secure firearm storage). To reach firearm owners with LMS messaging, researchers have developed suicide prevention training content which can be incorporated into firearm training courses. However, no study to date has evaluated impact of such training on firearm course students’ subsequent knowledge, attitudes, and openness related to secure firearm storage. Thus, the current study sought to examine both the feasibility and acceptability of a LMS-focused suicide prevention training module among firearm course students, as well as the impact of this module on students’ secure firearm storage-related knowledge, attitudes, and openness.

**Methods:**

Firearm instructors (*N* = 6) and students in firearm classes (*N* = 83) were recruited to participate. Students were invited to complete voluntary, anonymous pre- and post-surveys during courses they attended that were led by these instructors. Instructors and students were also invited to complete a brief qualitative interview.

**Results:**

Results indicated that firearm instructors and students in firearm courses found the module feasible and acceptable. Additionally, students’ knowledge about the relationship between firearms and suicide, openness to changing firearm storage practices, and endorsement of the importance of discussing firearms and suicide with fellow firearm owners, as well as willingness and confidence to do so, all significantly increased after viewing the module.

**Conclusions:**

These findings provide strong support for the use of such culturally competent LMS messaging as upstream suicide prevention in settings such as concealed carry courses.

**Supplementary Information:**

The online version contains supplementary material available at 10.1186/s40621-024-00526-0.

## Background

In 2021, suicide was the 11th leading cause of death in the United States (US), accounting for over 48,000 deaths (American Foundation for Suicide Prevention 2024). Certain groups, such as Veterans, experience a higher suicide rate than the general US population. Indeed, Veterans account for about 14% of all suicide deaths among US adults and have an adjusted suicide rate that is 72% higher than that of adult civilians (U.S. Department of Veterans Affairs 2023). In addition, firearms are the most commonly utilized method for suicide among both civilian adult (55%) and Veteran (72%) populations, and over half (54%) of all firearm-related deaths can be attributed to suicide (U.S. Department of Veterans Affairs 2023; Centers for Disease Control and Prevention, National Center for Injury Prevention and Control 2024; Gramlich 2023). Given their 90% case fatality rate, firearms are the most lethal method for suicide (Cai et al. 2022), highlighting the importance of addressing firearm suicide prevention in the US and among Veterans specifically.

Numerous US government agencies and experts in suicide research have emphasized the importance of upstream suicide prevention: efforts to target malleable risk factors and increase protective factors before acute suicide risk develops (Iskander and Crosby 2021; The White House 2021). Indeed, suicidal thoughts are difficult to predict as they are often episodic and dynamic, with swift onset and variable duration (Kleiman and Nock 2018; Coppersmith et al. 2023). Furthermore, previous research contends that mere accessibility to a firearm in the home is associated with elevated suicide risk and most firearm owners store at least one firearm in a highly accessible manner (Kellermann et al. 1992; Shenassa et al. 2004; Anestis et al. 2023). Thus, approaches that seek to increase environmental safety prior to a suicidal crisis may hold the most promise for broad suicide prevention. An important example of this type of approach is lethal means safety (LMS), an empirically-supported intervention that seeks to put time and distance between an individual who may be going through an emotional/suicidal crisis and the selected method for suicide (Henn et al. 2019).

Despite robust preliminary support for the effectiveness of LMS in reducing risk for firearm suicide (Anestis et al. 2021a; Simonetti et al. 2021), LMS messages often fail to reach the intended audience: firearm owners (Rowhani-Rahbar et al. 2018). One pathway to reach this group is through firearm safety or concealed carry courses in the community (Consolino and Yarvis 2022). Notably, just over half of all US states require proof of formal firearm training to purchase a firearm or obtain a concealed-carry permit, and many such trainings include information about secure firearm storage (typically in the context of preventing unauthorized or child access to firearms; RAND 2023). However, available research indicates that such training courses do not necessarily result in increased use of secure firearm storage (RAND 2023). Furthermore, almost no US states mandate the inclusion of suicide prevention in their firearm training courses (United States Concealed Carry Association 2024), and a 2015 nationally-representative survey revealed only 15% of firearm owners had learned about suicide prevention in the context of firearm training (Rowhani-Rahbar et al. 2018). Similarly, a separate examination of 20 firearm safety courses across multiple Northeastern states found that only 10% of firearm instructors integrated suicide prevention content into their training (Hemenway et al. 2019). This suggests that, within firearm training contexts, suicide risk is not frequently brought to the attention of firearm owners as a potential source of motivation for engaging in secure firearm storage, highlighting both a gap in knowledge and an opportunity to utilize such courses as a pathway to LMS education.

This approach was explored in a recent study by Barber et al. (2019), who helped create a suicide prevention and LMS module, which included a PowerPoint presentation and a brief video demonstrating how a firearm instructor could teach the material. The module encouraged firearm owners to routinely store firearm(s) securely and consider alternative measures for suicide prevention, such as temporary out-of-home storage (Harvard T.H. Chan School of Public Health 2024). The module was shared with over 1000 firearm course instructors, and 66% reported they would like to implement it in their classes. The module was subsequently published by the Utah Department of Public Safety in 2016 and was made a mandatory component of training for Utah concealed firearm permits in 2019 (Barber et al. 2019). This pioneering work demonstrated interest in and commitment to suicide prevention training among firearm instructors but was not without limitations. Specifically, feedback on the module was not collected from firearm course students, potentially overlooking crucial perspectives from the intended audience. Moreover, the brief video developed by Barber et al. (2019) was primarily designed as a training tool for instructors regarding how to deliver LMS content, rather than as a standalone means of conveying LMS content to firearm course students. The video’s focus on Utah-specific suicide statistics also limited its applicability and relevance to firearm owners and instructors in other states (Barber et al. 2019; Houtsma et al. 2023).

Against this backdrop, Houtsma et al. (2023) collaborated with local civilian and Veteran firearm owners to adapt the module for use in Louisiana firearm safety and concealed carry courses. To address limitations of prior research, an initial round of focus groups was conducted with firearm owners and instructors. Focus group participants were shown the module developed by Barber et al. (2019); (i.e., PowerPoint presentation and informational instructor video), followed by questions and discussion designed to elicit feedback on ways to improve the training module and make material more relevant to Louisiana firearm culture. Based on their feedback, Houtsma et al. (2023) developed an adapted module, titled “Saving Lives Together” (VISION 2024). A second round of focus groups with firearm owners and instructors found the adapted module to be unanimously acceptable and feasible for instructors to incorporate in their courses. Participants reported their suggestions had been adequately addressed, and qualitative analysis indicated that module adaptations increased acceptability (Houtsma et al. 2023).

Although these findings suggest that such a module *can* be used in firearm safety and/or concealed carry courses, they do not elucidate the impact that such a module has on firearm owners (Houtsma et al. 2023). It is critical to evaluate whether culturally-competent LMS messaging can increase knowledge about the risks of firearms and suicide, and positively impact attitudes and openness to secure storage and related behaviors. Thus, with the present study, our goals were to (1) pilot the module developed by Houtsma et al. (2023) in firearm safety and concealed carry courses in Louisiana, (2) evaluate the feasibility and acceptability of the module among firearm course students and instructors, and (3) evaluate impact of the module on relevant outcomes (e.g., knowledge about the risks of firearms and suicide, attitudes towards conversations about firearms and suicide prevention, and openness to change firearm storage practices) among firearm course students. It was hypothesized that after viewing the learning module, firearm course students would demonstrate increased (1) knowledge about the risks of firearm suicide, (2) positive attitudes (e.g., perceived importance, willingness, and confidence) surrounding conversations about firearms and suicide prevention, and (3) openness to changing firearm storage practices. It was also expected that firearm course students and instructors would find the module feasible and acceptable. If findings from the current study align with hypotheses, it would provide strong support for the use of such culturally-competent LMS messaging as upstream suicide prevention in settings such as firearm safety and concealed carry courses.

## Methods

### Design

We report this study in line with the Revised Standards for Quality Improvement Reporting Excellence (SQUIRE 2.0), found in Additional File 1 (Ogrinc et al. 2016). This seven-month pilot implementation trial was part of a larger quality improvement project (i.e., Veteran-Informed Safety Intervention & Outreach Network [VISION]; see Houtsma et al. 2023 for more details), which was deemed exempt from institutional review by the local Institutional Review Board. The study used a mixed methods design, integrating both quantitative and qualitative approaches for data collection and analysis.

### Participants

Participants were firearm instructors (*N* = 6) and students in concealed carry firearm classes (*N* = 83). Firearm instructors were recruited through VISION, and most (*n* = 4) participated in the initial development of the learning module in previous focus groups (Houtsma et al. 2023). Demographic information was not collected from firearm instructors. Students were invited to complete voluntary, anonymous pre- and post-surveys during courses they attended that were led by these instructors. All participants who opted to engage in the study completed the pre- and post-surveys. To obtain a concealed handgun permit in Louisiana, it is required that the applicant demonstrate competence with a handgun during their training, including live range shooting (Louisiana State Police 2024). Therefore, it is presumed that most, if not all, students were firearm owners at the time of their participation in this study. A majority of students who completed the surveys were male, White, and Non-Hispanic. A minority (6%) indicated they themselves were Veterans, and a majority of students (80.7%) reported they are family members of military Service Members or Veterans. Full student demographic information can be found in Table 1*. Student Demographic Information.*

**Table 1:** Student demographic information

*N* = 83	
*M*_age_(SD)	40.78 (14.92)
Gender	
Male	71.1
Female	28.9
Race	
White	72.3
Black	14.5
Asian/Pacific Islander	13.3
Ethnicity	
Non-Hispanic/Latinx	95.2
Hispanic/Latinx	2.4
Marital status	
Married	61.4
Never married	30.1
Divorced/separated	8.4
Education level	
Less than HS diploma/GED	1.2
HS diploma/GED	51.8
Vocational school	8.4
College degree	30.1
Graduate school degree	8.4
Military Service (self)	
Yes	6.0
No	94.0
Military service (family)	
Yes	80.7
No	19.3

Values displayed in percentages; HS = High School; GED = General Educational Development

### Procedures

#### Introductory instructor meeting

The first author held a 90-min introductory meeting with the firearm instructors who agreed to participate in pilot implementation. This involved re-orienting instructors to VISION, the parent project described above, introducing the instructors to the suicide prevention learning module (i.e., showing the video and PowerPoint presentation), and discussing the specific procedures and instructor responsibilities during the seven-month pilot implementation.

#### Intervention

The suicide prevention learning module is described in detail elsewhere (Houtsma et al. 2023) and is available online (VISION 2024). In short, the module includes a brief video (approximately 6 min), wherein two Veteran firearm owners present information on local and national suicide statistics, warning signs of suicide risk, evidence supporting LMS as an effective approach to suicide prevention, personal stories about firearm suicide risk, and local and national suicide prevention resources. As an alternative or in addition to the video, there is a PowerPoint presentation covering the same topics.

#### Data collection

##### Anonymous survey data

Instructors were mailed packets of blank surveys pre-sorted into envelopes labeled with participant numbers. Each envelope contained a pre- and post-intervention survey, to be completed by students before and after viewing the module. Instructors were advised to distribute one envelope to each student interested in participating. Prior to sharing the suicide prevention learning module, instructors guided students to complete the pre-intervention survey. Instructors then shared the suicide prevention learning module in their course. Finally, instructors guided students to complete the post-intervention survey, place both completed surveys back into the envelope, and seal it. Firearm instructors then collected the sealed envelopes, and these were returned to the study team.

##### Follow-up interviews

Students were invited to participate in a follow-up interview approximately one-month after viewing the learning module. Individuals who self-referred were engaged in a 15–30-min, telephone interview with a member of the study team. The study team member took notes on students’ responses and the interviews were not audio-recorded. Firearm instructors were also contacted to participate in a brief, audio-recorded telephone exit interview after data collection was completed. Instructor interviews were transcribed by a member of the study team for qualitative analysis.

##### Measures

The following measures were developed for the purposes of the current study, with the exception of the Openness to Change measure, which has been utilized in prior research (Anestis and Houtsma 2019). See Additional file 2 for a full listing of items.

##### Knowledge

Knowledge regarding firearm suicide was assessed through a 4-item measure, with response options including “True”, “False”, and “Not Sure.” Items assessed knowledge about the proportion of firearm deaths in Louisiana attributed to suicide, whether it is possible to identify risk for suicide, the brief amount of time between suicidal thoughts and action, and preventative actions for suicide (e.g., “Putting time and space between a suicidal person and a firearm can decrease risk of suicide”). Total scores were created by assigning a “1” to correct responses across items and a “0” for incorrect responses or responses of “Not Sure.” This resulted in total scores ranging from 0 to 4. Given items in this measure were assessing knowledge across different concepts, it was deemed inappropriate to calculate internal consistency.

##### Attitudes: importance, willingness, and confidence

A 3-item measure was used to assess students’ perceived importance, willingness, and confidence in discussing mental health and suicide prevention with other firearm owners (e.g., “It is important for firearm owners to talk about mental health and suicide prevention”). Response options ranged from 0—“Strongly Disagree” to 4—“Strongly Agree,” with total scores ranging from 0 to 12. Internal consistency for this measure was strong at pre- and post-learning module exposure (α = 0.86 and α = 0.78, respectively).

##### Openness to change

An 11-item measure used in prior research (Anestis and Houtsma 2019) assessed students’ openness to changing a variety of firearm storage practices in the context of their own OR a loved one’s suicide risk (e.g., “Are you open to temporarily storing your firearm(s) away from your home to prevent a suicide attempt by yourself?”). Response options ranged from “Not at all Open” to “Extremely Open,” with an option to select “Not applicable—firearms already stored this way”, across a 5-point Likert scale. This latter option was categorized as the highest possible score for a single item, as it indicated the individual was already using this secure storage practice. The total score for this measure ranged from 0 to 55. Internal consistency for this measure was very strong at pre- and post-learning module exposure (α = 0.96).

##### Acceptability

Acceptability of the learning module was evaluated using a 2-item measure which inquired as to the perceived appropriateness of the learning module in a firearm training course, as well as their likelihood to recommend the module to other firearm owners. Response options ranged from 0—“Strongly Disagree” to 4—“Strongly Agree,” with total scores ranging from 0 to 8. Total scores above the middle of the scale (i.e., above the mean score of 4) would suggest the module was acceptable. The measure demonstrated very strong internal consistency (α = 0.95).

##### Semi-structured interviews

Interviews with students explored whether messaging from the suicide prevention learning module had a sustained impact, was acceptable in the setting of a firearm training course, and had resulted in any changes to relevant behaviors (e.g., firearm storage). Exit interviews with instructors sought to identify barriers and facilitators to incorporating the learning module into their classes, acceptability of the module, and future plans regarding use of the module in their classes. See Additional File 2 for interview guides.

#### Compensation

Instructors were compensated $200 for attending the 90-min introductory orientation meeting, as well as $200 for each class in which they incorporated the module and facilitated data collection. Of note, no instructor incorporated the module into more than five courses. Students who completed the pre- and post-surveys during their firearm class were not compensated due to anonymity and feasibility issues, but those who self-referred for follow-up interviews were compensated $50 for their time and effort.

### Data analytic plan

#### Quantitative analyses

Paired samples *t*-tests were conducted to evaluate the impact of the suicide prevention learning module on students’ knowledge about firearms and suicide, attitudes towards discussing firearms and suicide with fellow firearm owners (i.e., perceived importance, willingness, and confidence), and openness to changing firearm storage practices. To evaluate acceptability of the learning module, mean scores on the 2-item acceptability measure were examined descriptively.

#### Qualitative analyses

Two separate theoretical thematic analyses were conducted using data from qualitative interviews with 1) instructors (*n* = 4), and 2) students (*n* = 12) to further evaluate feasibility, acceptability, and impact of the learning module. The study’s research questions (e.g., “Is it acceptable to include a suicide prevention module in a firearms training course?”) guided the analysis. Specifically, a realist framework and semantic approach to theme identification were employed, allowing the researchers to use the interview questions and participants’ explicit answers as the foundation for theme identification, as opposed to attempting to identify underlying ideologies (Braun and Clarke 2006; Lochmiller 2021). To conduct these analyses, two authors (C.H. and K.M.), engaged in iterative review and discussion of the interview transcripts. The authors independently coded, categorized, and identified themes from these categories. Each step was followed by discussion to resolve discrepancies (Lochmiller 2021).

## Results

### Quantitative analyses

A total of 83 students completed the pre- and post-surveys. Results of a paired-samples *t*-test revealed there was a significant increase in knowledge about firearms and suicide among firearm training course students from pre- (*M* = 1.90, *SD* = 0.95) to post-intervention (*M* = 3.85, *SD* = 0.42), *t*(79) = − 16.54, *p* < 0.001, *d* = 1.85. A paired samples* t*-test also demonstrated a significant increase in positive attitudes (i.e., importance, willingness, and confidence) towards discussing mental health and suicide prevention with fellow firearm owners from pre- (*M* = 7.70, *SD* = 2.91) to post-intervention (*M* = 9.31, *SD* = 2.24), *t*(81) = − 9.58, *p* < 0.001, *d* = 1.06. A final paired samples *t*-test revealed a significant increase in openness to changing firearm storage from pre- (*M* = 30.20, *SD* = 10.27) to post-intervention (*M* = 36.83, *SD* = 7.95), *t*(79) = − 9.12, *p* < 0.001, *d* = 1.02. Students’ scores on the measure of module acceptability were above the mean of 4 (*M* = 7.19, *SD* = 1.08).

### Qualitative analyses

#### Students

A total of 12 students completed follow-up qualitative interviews. In total, the authors identified 44 codes, 7 categories, and 4 themes.

##### Theme 1: students retained the main message and learned about suicide risk more broadly

All students recalled learning about suicide prevention in their firearm training course, most often remembering the module video, followed by the PowerPoint slides and instructor-led discussion. Some students had difficulty recalling a specific main message; however, most identified *temporarily removing firearms in the event of suicide risk* as the main message of the module. Many students recalled other information from the module such as awareness of signs of suicide risk, particularly within the context of firearm ownership, talking to someone and asking for help when facing mental health challenges and/or suicidal thoughts, and being available to provide support to others. Some students reported previous awareness of the connection between suicide risk and firearms, and therefore did not believe the module impacted their knowledge of the topic. However, many students expressed that they learned new information as a result of viewing this module, with the most impactful information being statistics about firearm suicide, the importance of raising awareness about the risks of firearm suicide, specific ways to practice secure firearm storage, and how to recognize warning signs of suicide risk.

*Category: Format*—“(We) watched a video outlining statistics and then had a very honest discussion afterwards.”

*Category: Main messages*—“Main ideas of calling if you need help and taking guns out of homes of those who are at risk of suicide.”

*Category: Important take-aways*—“The awareness is a very important message to anyone that owns a firearm.”

*Category: Shed new light*—“eye-opening”, “mind-blowing”, “enlightening”, “I felt somewhat enlightened.”

##### Theme 2: the module had an impact on some students' thoughts, emotions, and behavior

Some students reported feeling concerned about the prevalence of firearm suicide, whereas others expressed a sense of positivity related to spreading awareness and the willingness of others to provide help. Notably, some participants indicated that their emotions remained largely unaffected by the module. With regards to the impact of the module on students’ thoughts and behaviors, three students indicated they began storing their firearm(s) more securely. A few students expressed willingness to temporarily remove firearms from the home if someone in their household became at risk for suicide, and others indicated they would be more vigilant for warning signs of suicide risk. A significant number of students said they had not changed their firearm storage behaviors since attending the firearms class, though several of them noted that they already stored their firearm(s) securely prior to viewing the module.

*Category: Emotional impact—*“Felt good knowing that others are aware and that there is a community available if in need.”

*Category: Important takeaways—*“I think it changed how I see suicide risk because at first, I didn’t know it was a big thing when you’re dealing with firearms, and now, it changed my view that I need to take having a firearm more seriously because it’s not a game.”

*Category: Behavioral changes—*“It just showed me another way to store my firearm and taught me not to rely on firearms that much. Now, I don’t keep it lying around, especially if I’m down.” When asked if they discussed the materials with anyone else “Yes, with a friend”.

##### Theme 3: the majority found the module acceptable, although some concerns were highlighted

Overall, students found the module to be acceptable, with several emphasizing the importance and relevance of including it in firearm safety courses. Many stated that the setting was appropriate and the module facilitated helpful discussion. Concerns were noted by a minority of students, including the belief that the module was not relevant to each student, the belief that the video lacked sufficient emotional impact, and the belief that the module could trigger suicide risk.

*Category: Acceptability—*“Very acceptable, something that everyone needs to be aware of and that is often taken for granted.”

*Category: Emotional *impact—“It was great listening but could have used more examples of families or friends to bring the whole message together. It lacked impact.”; “This material could be triggering to some, and those people could lose their life due to it being talked about.”

#### Instructors

A total of four instructors completed exit interviews. In total, the authors identified 39 codes, 6 categories, and 3 themes.

##### Theme 1: the module is relevant and easy to incorporate

Instructors reported the video format and brevity of the module facilitated incorporation into their courses. Two of the instructors integrated the module into the child access prevention section, while the other two presented it at the end of the course. Instructors noted that student-led conversations about secure firearm storage and suicide prevention occurred in response to the module, demonstrating the relevance and acceptability of the material. Importantly, some barriers and challenges were identified. Specifically, some instructors believed that the module's focus on Veterans was a minor barrier, as not all students may feel the information was relevant to them. Additionally, one instructor expressed that incorporating a new element into his course was a slight challenge.

*Category: Functionality—*"It [video] conveyed the message really well, really easily. It was not a whole lot of question marks there, so I would say that was maybe the easiest element of all."

*Category: *Student* reactions*—“I think everyone was able to make kind of direct connections…I think that’s what gave it so much credibility.”

*Category*: *Barriers, challenges, and suggestions*—“The only disconnect I would say is with the folks who were not Veterans…because it almost sounded like it was something specific to Veterans and suicide prevention and mental health issues with Veterans.”

##### Theme 2: the module engaged students and added value to the course

Instructors expressed that the module facilitated open conversations about suicide, heightened awareness of suicide and prevention among students, and provided valuable insights into how to assist others in crisis. During these discussions, students and instructors shared their personal experiences, adding substantial value to the course. The incorporation of a video component effectively increased class engagement and motivation, which was evident to instructors through students’ body language and participation in conversations. Instructors emphasized the positive impact of the module, suggesting every student benefitted from it. Instructors noted key module components that helped to achieve positive outcomes, including impactful statistics and use of visuals to convey the gravity of the issue. Additionally, instructors noted that group discussions were integral to the module’s impact. Instructors reported that their students appeared satisfied with the information in the module.

*Category: Module consequences*—“I found it opened up a whole other line of talking. Suicide is a very difficult area to talk about with people usually, and this opened up that discussion, which was really good.”

*Category*: *Key module components—“*That was a big eye-opener for a lot of people whenever they broke down the statistics of who this affects, so that was very good, very beneficial information there.”

*Category: Student reactions*—“Everyone was pretty satisfied with the information that they learned.”

##### Theme 3: instructors feel empowered and motivated to spread the word

Instructors reported motivation to share the module content with others. Specifically, all instructors expressed plans to continue showing the module in their courses and stated they would likely recommend it to their peers. Indeed, some instructors showed their passion by presenting or describing plans to present the module to other groups (e.g., self-defense class). Furthermore, instructors suggested that the study team explore additional avenues for dissemination, such as posting the module on social media and requesting to include it in state curriculum for Louisiana concealed carry courses.

*Category: Instructors’ intentions and actions—*“I’m also a member of another group, and I have suggested this module to them to incorporate.”

*Category: Barriers, challenges, suggestions—“*I believe thoroughly it should be part of the required teaching syllabus for every concealed carry instructor.”

## Discussion

The current study demonstrated that the suicide prevention learning module developed by Houtsma et al. (2023) can be implemented in concealed carry courses, is viewed as feasible and acceptable among students and instructors, and leads to meaningful change among students who view it. These findings support the use of suicide prevention content in Louisiana concealed carry courses, and may offer preliminary support for use of such content in firearm safety courses, thereby highlighting an important avenue through which to reach firearm owners before risk of suicide develops. This study builds upon previous literature by examining the acceptability and impact of a suicide prevention learning module directly among students of concealed carry courses.

According to findings from both the quantitative and qualitative data, the module was viewed as acceptable. Instructors noted that the module added value and reported the module opened up important conversations about firearm suicide prevention and secure storage in their classes. Instructors’ intentions and behaviors aligned with this feedback, given that many planned to or already had shared the module with other individuals. Students largely agreed that the module was relevant to include in concealed carry courses and most expressed having learned new information. A minority of students noted concern that the topic of suicide may be irrelevant to some students or could lead to an increase in suicide risk among others. Although it is commonly believed that asking about or discussing suicide can increase subsequent suicide risk, this is not supported by research; in fact, some studies have found that such conversations can actually reduce suicidal ideation (Dazzi et al. 2014). Despite some instructors’ concerns, the module’s use of Veteran speakers and inclusion of Veteran-specific statistics did not appear to negatively impact students’ perceptions about the relevance of the material, given that none offered negative feedback regarding the Veteran speakers or statistics and one student offered positive feedback about these features. This aligns with prior research showing that Veteran and non-Veteran firearm owners perceive Veterans to be credible LMS messengers (Pallin et al. 2019; Anestis et al. 2021b). Indeed, although only a small proportion of the sample in the current study identified as Veterans, the majority of students identified as a family member of a Service Member or Veteran. The use of Veteran speakers may have been relatable to such students and, importantly, family members may play a role in helping Veteran loved ones to adopt more secure firearm storage practices (Betz et al. 2023).

Importantly, results supported the impact of the module across several areas related to suicide prevention. Specifically, students’ knowledge about the relationship between firearms and suicide, as well as their knowledge about suicide risk in general significantly increased after viewing the module. Understanding these facts is crucial, as research shows that misinformed beliefs about firearms and suicide are associated with less secure firearm storage practices and lower openness to changing firearm storage practices (Anestis et al. 2018). Indeed, students in the current study became more open to changing firearm storage practices after viewing the module, perhaps suggesting that correcting misinformed beliefs can increase openness to alternative storage practices. Finally, students exhibited higher endorsement of the importance of discussing firearms and suicide with fellow firearm owners, as well as willingness and confidence to do so, after viewing the module. Notably, all of the aforementioned changes were robust, with effect sizes in the strong or very strong range (Sawilowsky 2009; Cohen 1988). Furthermore, students were able to recall the main message of the module (i.e., securely storing or temporarily removing firearms from the home during times of suicide risk can save lives) one month after their concealed carry course, which indicates that the module effectively communicated the importance of secure firearm storage in the context of suicide risk. Some students had even engaged in behavior change, including talking to their family and friends about the content of the module or increasing the security of their firearm storage.

The current study has several limitations. Outcome variables of interest were assessed among a small group of instructors and students. Moreover, all participants were Louisiana residents, all were participating in concealed carry courses, and the sample was fairly homogenous regarding demographic characteristics. Results presented here may not be representative of all Louisiana firearm owners or firearm owners from other US states. Future studies should evaluate outcomes among varied, larger, and more diverse samples, such as samples of women and Veteran firearm owners or those taking firearm safety courses as opposed to concealed carry courses, to ensure findings capture important differences. Second, only firearm course students interested in the research study completed survey measures, and students referred themselves to participate in the optional one-month follow-up interview. This self-selection bias may further limit the generalizability of results. Additionally, many of the outcome measures used in the current study were created by the authors and not psychometrically tested, meaning validity and reliability of these measures is unknown. Notably, a bill was passed in Louisiana just after the study concluded allowing honorably discharged Veterans and currently military Service Members to carry a concealed weapon without obtaining a permit or undergoing further training (Louisiana State Legislature 2022). As such, future studies will need to explore alternative routes to ensure this module or similar messages reach Louisiana Veterans and Service Members, such as through Veterans Service Organizations. Lastly, four out of six of the firearm course instructors participating in this study also assisted in the original adaptation of the module in Houtsma et al. (2023) and may have had a more positive bias towards the implementation of and outcomes related to the module.

## Conclusions

Despite these limitations, the present study expanded upon previous efforts (Barber et al. 2019; Houtsma et al. 2023) to successfully incorporate a suicide prevention module in the curriculum for Louisiana concealed carry courses. Not only do findings indicate the module is acceptable and can be feasibly integrated into concealed carry courses, but also that this intervention positively impacts knowledge, attitudes, and openness to behaviors related to suicide prevention and LMS. Finally, the broader community may benefit from participating in this learning module, so future work should consider more widespread implementation. Given that suicidal behaviors are difficult to predict and suicide attempts involving firearms are often lethal, interventions promoting upstream suicide prevention are imperative. This study supports this public health approach and provides evidence that these vital messages can reach the intended audience and may lead to meaningful changes relevant to suicide prevention.

### Supplementary Information

Additional file 1.

Additional file 2.

## Data Availability

The first author, CH, can make data available upon reasonable request.

## Abbreviations

LMS: Lethal means safety
VISION: Veteran-informed safety intervention & outreach network
US: United States
