# Supplementary material for: Impact of a suicide prevention learning module for firearm training courses in Louisiana
Source: Inj Epidemiol. 2024 Sep 2;11:41. doi: 10.1186/s40621-024-00526-0 (PMC11367988; doi:10.1186/s40621-024-00526-0)
Supplement: Supplementary file 2 — Additional file 2. [file 40621_2024_526_MOESM2_ESM.docx]

Additional file 2: Supplementary Material

**Student Assessments**

**Knowledge**

1. Nearly half of all firearm-related deaths in Louisiana are suicides. *Answer: True*
2. There is no way to identify whether someone is struggling and at risk of suicide. *Answer: False*
3. The time between thinking about attempting suicide and acting on those thoughts is often brief. *Answer: True*
4. Putting time and space between a suicidal person and a firearm can decrease risk of suicide. *Answer: True*

Response options: True, False, Not Sure

**Openness to Change**

1. Are you open to temporarily holding the firearms of a friend or family member to prevent their suicide attempt?
2. Are you open to temporarily storing firearm(s) away from your home to prevent a suicide attempt by yourself?
3. Are you open to temporarily storing firearm(s) away from your home to prevent a suicide attempt by a loved one or someone who lives with you?
4. Are you open to storing your firearm(s) in a safe to prevent a suicide attempt by yourself?
5. Are you open to storing your firearm(s) in a safe to prevent a suicide attempt by a loved one or someone who lives with you?
6. Are you open to storing your firearm(s) with a locking device (e.g., trigger or cable lock) to prevent a suicide attempt by yourself?
7. Are you open to storing your firearm(s) with a locking device (e.g., trigger or cable lock) to prevent a suicide attempt by a loved one or someone who lives with you?
8. Are you open to storing your firearm(s) unloaded to prevent a suicide attempt by yourself?
9. Are you open to storing your firearm(s) unloaded to prevent a suicide attempt by a loved one or someone who lives with you?
10. Are you open to storing your firearm(s) separate from ammunition to prevent a suicide attempt by yourself?
11. Are you open to storing your firearm(s) separate from ammunition to prevent a suicide attempt by a loved one or someone who lives with you?

Response options: Not at all open, Somewhat open, Moderately open, Very open, Extremely open, Not applicable – firearms already stored this way

**Importance, Willingness, and Confidence**

1. It is important for firearm owners to talk about mental health and suicide prevention.
2. I am willing to talk to other firearm owners about mental health and suicide prevention.
3. I am confident in my ability to discuss mental health and suicide prevention with fellow firearm owners.

Response options: Strongly disagree, Disagree, Neither agree nor disagree, Agree, Strongly Agree

**Acceptability**

1. I think it is appropriate to have suicide prevention materials like this in my firearm training course.
2. I would recommend this suicide prevention module to other firearm owners.

Response options: Strongly disagree, Disagree, Neither agree nor disagree, Agree, Strongly Agree

**Follow up interview Guides**

**Student Interview Guide – One-Month Follow-Up**

Thank you for participating in this study and agreeing to talk with me about your experience with suicide prevention materials that were included in the concealed carry or firearm safety course you took from [insert firearm course instructor name] about a month ago.

During the class, you answered a few questions about your beliefs and thoughts about firearms and suicide. Today, I’d like to learn more about your thoughts on the suicide prevention training that was included in your class.

Before we begin, I would like to talk about the interview process. You can choose to skip any question if you feel uncomfortable. Also, I will be typing while we talk so that I can capture your comments. I will not include any identifying information (e.g., names, cities, etc.) in what I type out so that your comments remain anonymous. Do you have any questions before we begin?

1. Please describe what, if any, suicide prevention training you received during your course.

Probes:

- Were you shown a video or PowerPoint slides during your class? What do you recall your instructor saying about suicide prevention?

1. How would you describe the main message of the suicide prevention training?

Probes:

- What message(s) or information do you recall from the suicide prevention training?

1. How acceptable was it to you, to receive suicide prevention training in your concealed carry course or firearm safety course?

Probes:

- Was this an appropriate setting to receive this information?
- How did it make you feel to learn about this in your class?

1. How did the suicide prevention training impact you after leaving the class?

Probes:

- Did you discuss the lessons or materials with anyone else? Family, friends, fellow firearm owners?
- Did you change the way you store or handle your own firearms?
- How has it impacted the way you think about firearms and suicide risk?

1. Are there any other thoughts or information you’d like to share with me about the suicide prevention materials that were shared with you in your firearm class?

Thanks so much for your time and for sharing your insights. We will be sending you a $50 gift card in the mail as a thank for you speaking with us. I’d like to check that I have the correct address for us to mail you the gift card. The address I have is____________________. Is that correct?

Thank you.

**Instructor Exit Interview Guide**

1. What got in the way of incorporating the suicide prevention module?

Probes:

- What was hard about incorporating the suicide prevention module?

1. What made it easier to incorporate the suicide prevention module?
2. How much value did the suicide prevention module add to the class?
3. What parts of the suicide prevention module seemed most effective?

Probes:

- How could you tell that (that part) was effective?

1. What parts seemed less effective?

Probes:

- How could you tell that (that part) was less effective?

1. How did you choose to show the suicide prevention module?

Probes:

- Why?

1. What did your students think about the suicide prevention module?

Probes:

- What specific feedback did you get?

1. Do you plan to continue incorporating the suicide prevention module into your classes?

Probes:

- If yes, how do you plan to incorporate it?
- If no, why not? How likely would you be to recommend this module to other instructors? Why or why not?
